# How parental mediation relates to digital device use in primary and secondary school students: evidence for the statistical mediation of parent–child interaction quality

**DOI:** 10.3389/fpsyg.2026.1811092

**Published:** 2026-05-08

**Authors:** Mingling Zhao, Junhui Zhu, Junwu Xu, Ling Wang, Tongxu Luo, Sirong Song

**Affiliations:** 1 Chengdu Polytechnic, Chengdu, China; 2 Tianfu New Area Education Science Research Institute, Chengdu, China; 3 West Campus of Chengdu Experimental Foreign Languages School, Chengdu, China; 4 Sichuan Tianfu New Area Experimental Primary School, Chengdu, China

**Keywords:** digital device use, indirect association, parental mediation strategies, parent–child interaction, primary and secondary school students

## Abstract

**Introduction:**

Digital device use has become increasingly common among children and adolescents, making it important to understand how parental mediation strategies relate to students’ device-use behaviors and the family relational context in which such behaviors occur.

**Methods:**

This cross-sectional, multi-informant questionnaire study examined associations among parental mediation strategies, parent–child interaction quality, and students’ digital device use. Participants were 1,235 paired primary and secondary school students in Grades 4–8 and their parents from Tianfu New Area, Chengdu, China. Descriptive statistics, group comparisons, correlation analyses, mediation models, and path analysis were used to examine the focal variables.

**Results:**

Digital device use was widespread and often began in early childhood. Restrictive mediation was associated with lower device-use time, less entertainment-oriented content, and less intensive use habits, whereas co-use and active mediation were positively associated with better parent–child interaction quality. Parent–child interaction quality showed significant statistical mediation in the associations of co-use, active mediation, and restrictive mediation with overall digital device use.

**Discussion:**

Because the data were cross-sectional, the findings should be interpreted as relational rather than causal. Overall, the results highlight the family relational context in which parental mediation is linked to children’s digital device use.

## 1 Introduction

### 1.1 Research background

Over the past two decades, digital devices have become routine tools in the everyday lives of children and adolescents, particularly for learning, entertainment, and social contact (Livingstone and Helsper, 2007; Rideout et al., 2010). At the same time, the literature suggests that heavier or poorly regulated screen-based engagement may co-occur with lower sleep quality, weaker emotional regulation, attentional difficulties, and less favorable academic adjustment, although the magnitude and direction of these associations vary across contexts and types of use (Hale and Guan, 2015; Kuss and Griffiths, 2017; Twenge and Campbell, 2018).

Within family life, parental responses to children’s digital device use have therefore become a major educational concern. Prior research indicates that parental mediation is not a single practice but a cluster of strategies - such as co-use, active guidance, restriction, monitoring, and technical control - that may relate differently to children’s media experiences and to the relational climate of the family (Valkenburg et al., 1999; Valkenburg et al., 2013). For this reason, it is important to examine not only device use itself, but also the parent–child interaction context in which that use is negotiated.

### 1.2 Research questions and objectives

Although the literature has documented many correlates of children’s digital device use, fewer studies have examined how distinct parental mediation strategies are differentially associated with students’ use patterns and how parent–child interaction quality may operate as a relational link within those associations. Existing work has often emphasized outcomes of screen use itself, while paying comparatively less attention to the family processes through which parental mediation and children’s everyday device practices become connected.

Accordingly, the present study examines associations between parental mediation strategies and digital device use behaviors among primary and secondary school students, with particular attention to the statistical mediation of parent–child interaction quality. The study addresses three questions: (a) How are parental mediation strategies associated with students’ digital device use behaviors? (b) Does parent–child interaction quality show a statistically significant indirect pathway within these associations? and (c) Do different parental mediation strategies display distinct association patterns with students’ digital device use behaviors?

### 1.3 Significance of the study

By examining the relationships between parental mediation strategies and parent–child interaction quality, this study extends existing research within the field of family education. The findings contribute to a more nuanced understanding of the potential pathway through which parental mediation strategies are associated with students’ digital device use behaviors and clarify the role of parent–child interaction quality as a mediating process, thereby offering new theoretical perspectives and empirical evidence for family education research.

From a practical standpoint, the study provides meaningful implications for parents, schools, and educational policymakers. The results may assist parents in understanding how strategies such as emotional support, reasonable restriction, and shared activities are associated with children’s digital device use. In addition, the findings may inform schools and policymakers in the development of home–school collaboration policies, promoting more effective coordination between families and schools in managing students’ digital device use.

## 2 Literature review and theoretical framework

### 2.1 Effects of digital device use on primary and secondary school students

Digital device use now occupies a visible place in the daily routines of many children and adolescents. Research has consistently shown that longer screen exposure and problematic engagement with social media, online games, or entertainment-oriented platforms are associated with a range of developmental outcomes, including sleep disturbance, emotional dysregulation, and weaker academic adjustment (Hale and Guan, 2015; Kuss and Griffiths, 2017; Twenge and Campbell, 2018). These findings do not suggest that all screen use is uniformly harmful; rather, they indicate that timing, content, duration, and family context all matter when interpreting children’s digital behaviors.

Adolescents who engage heavily in digital device use often exhibit lower academic performance and weaker emotional regulation, patterns that are closely related to self-control and attentional processes. Within this context, family education—and parental involvement in particular—has been increasingly recognized as a key contextual factor associated with adolescents’ digital device use behaviors.

### 2.2 The role of parental mediation strategies in adolescent behavior

Parents remain central actors in children’s digital lives. The parental mediation literature usually distinguishes among several strategies, including co-use, active mediation, restrictive mediation, monitoring, and technical restriction (Valkenburg et al., 1999; Valkenburg et al., 2013). These strategies differ in how directly they regulate children’s behavior and in the kind of interaction they require from parents. Consequently, they should not be treated as functionally equivalent when examining their associations with children’s digital device use.

Supportive mediation that involves explanation, discussion, and joint engagement may help preserve openness in family communication, whereas highly control-oriented practices may be associated with greater tension when they are perceived as rigid or one-sided (Koerner and Fitzpatrick, 2002; Noller and Bagi, 1985). Widyanto and Griffiths (2006) noted that although technical measures can restrict children’s access to digital devices, a lack of emotional support may be associated with increased emotional distance between parents and children (Widyanto and Griffiths, 2006).

### 2.3 The role of parent–child relationships in adolescent development

A substantial body of family research suggests that the quality of parent–child relationships is closely tied to children’s emotional adjustment, school adaptation, and self-regulation. Closeness, communication quality, emotional support, and manageable levels of conflict have all been associated with more adaptive developmental outcomes across adolescence (Noller and Bagi, 1985). Huimin (2015) demonstrated that parents’ perceptions of conflict were indirectly associated with adolescents’ peer relationships and social competence through parent–child relationship quality (Huimin, 2015). In addition, parent–child relationship quality has been shown to be associated with adolescents’ emotional regulation and self-efficacy, which are important for school adjustment.

Empirical studies have also indicated that parent–child relationship quality may function as an intermediary relational condition linking family processes with children’s behavioral outcomes. For example, parent–child relationship quality has been associated with school adjustment, emotion regulation, and problematic online behaviors in prior research (Shihua et al., 2015). Similarly, Tingdan et al. (2015) found that parent–child relationships were indirectly associated with adolescents’ online game addiction through self-esteem, highlighting the importance of relational processes in understanding problematic digital behaviors (Tingdan et al. 2015).

### 2.4 Theoretical framework

This study draws on family systems theory and parent–child communication theory to conceptualize the relationships among parental mediation strategies, parent–child interaction quality, and students’ digital device use behaviors.

Family systems theory views the family as an interconnected system in which family members mutually influence one another, and individual behaviors are shaped by broader patterns of family interaction. Within this framework, parental mediation strategies are understood as part of the family system that may be associated with children’s behaviors through their links with parent–child relational dynamics (Bowen, 1993; Cox and Paley 1997).

Parent–child communication theory emphasizes that open and effective communication serves as a foundation for healthy parent–child relationships and is associated with children’s self-regulation and emotional management. In the present study, this perspective provides a conceptual basis for understanding how parent–child interaction quality may function as a mediating process linking parental mediation strategies and students’ digital device use behaviors (Koerner and Fitzpatrick 2002; Noller and Bagi 1985).

### 2.5 Research gaps and contributions

Although previous studies have examined digital device use and parental mediation separately, fewer studies have considered how specific mediation strategies and day-to-day parent–child interaction quality may be connected within the same analytical framework. In addition, some studies have relied on broad parental control indicators without distinguishing among restrictive, active, shared, and technical forms of mediation.

By integrating family systems theory with parent–child communication theory, the present study treats parental mediation as a relationally embedded family practice rather than as a purely behavioral control tool. The contribution of the study lies in examining whether parent–child interaction quality statistically links parental mediation strategies with students’ digital device use in a single cross-sectional model, thereby refining the family-process perspective on children’s media use.

## 3 Study design and methods

### 3.1 Participants

This study adopted a cross-sectional, multi-informant questionnaire design. Participants were recruited from four middle schools and two primary schools in Tianfu New Area, Chengdu, Sichuan Province, China. Eligible students were enrolled in Grades 4–8, were able to complete the questionnaire independently or with routine classroom guidance, and had corresponding parental questionnaires available for matching. Cases were excluded when student-parent questionnaires could not be paired or when responses were substantially incomplete or failed quality screening. The final analytic sample comprised 1,235 student-parent pairs, including 619 boys and 616 girls. Available background variables included grade, place of residence, only-child status, primary caregiver, and household structure. More detailed background indicators, such as exact mean age, household socioeconomic status, and caregivers’ marital status, were not available for the present analyses and are therefore acknowledged as limitations.

### 3.2 Measures

#### 3.2.1 Digital device use behavior scale

Students’ digital device use behaviors were measured with an instrument adapted from the Digital-Screen Exposure Questionnaire (Kaur et al. 2021). The revised scale covered device access and type, frequency of use, weekday and weekend duration, major use content (learning/information vs. entertainment/social use), and conflict related to device use. Items were scored on Likert-type response formats, with higher scores indicating more frequent or more intensive digital device use and, where applicable, higher levels of device-related conflict. Dimension scores and an overall composite score were calculated by averaging relevant items. The original DSEQ has shown acceptable psychometric performance in prior validation work, and internal consistency screening was completed before the present substantive analyses.

#### 3.2.2 Parental mediation strategy scale

Parents’ mediation practices were assessed with the Perceived Parental Media Mediation Scale (Valkenburg et al. 2013), which covers co-use, active mediation, restrictive mediation, monitoring, and technical restriction. Parents indicated how often they adopted each strategy in everyday family settings. Mean scores were computed for each subscale, with higher values reflecting more frequent use of that mediation strategy. The source scale has demonstrated satisfactory reliability and construct validity, and in the present study each subscale was treated as a separate observed dimension after internal consistency screening.

#### 3.2.3 Parent–child interaction quality scale

Parent–child interaction quality was assessed using items adapted from the Parent-Adolescent Communication Scale (Sales et al. 2013). The revised measure focused on communication openness, emotional support, and conflict in daily parent–child exchanges. Negatively worded items were reverse-scored so that higher scores consistently reflected better interaction quality. A composite score was obtained by averaging item responses. Prior family and adolescent studies have used this instrument, and the present study employed it as an overall indicator of the relational quality perceived by students after consistency checking.

### 3.3 Data collection procedures

Data were collected through a cross-sectional questionnaire survey conducted in cooperation with participating schools in Tianfu New Area, Chengdu, Sichuan Province, China. Students completed the Digital Device Use Behavior Scale and the Parent–Child Interaction Quality Scale, whereas parents completed the Parental Mediation Strategy Scale, thereby reducing the degree to which all key constructs depended on a single informant. Participation was voluntary and anonymous. Written informed consent was obtained from parents or legal guardians, and student assent was obtained before questionnaire administration. No directly identifying personal information was retained in the analytic dataset.

### 3.4 Data analysis

Before substantive analyses, the dataset was screened for incomplete cases, apparent response-quality problems, and distributional assumptions. The normality of the major composite variables was examined using skewness and kurtosis statistics together with histogram and Q-Q plot inspection. Because no serious departures from normality were detected at the composite-score level, parametric analyses were retained. In addition, an exploratory Harman’s single-factor test was used as a diagnostic check for common method bias; the first unrotated factor did not account for a dominant share of the total variance, suggesting that common method bias was unlikely to fully explain the observed associations.

Descriptive statistics were used to summarize sample characteristics and the distributions of the focal variables. Group comparisons were conducted across gender, grade level, place of residence, only-child status, primary caregiver, and household structure using independent-samples *t* tests or one-way ANOVA, as appropriate. Pearson correlation analyses were then conducted to examine bivariate associations among parental mediation strategies, parent–child interaction quality, and students’ digital device use behaviors. To estimate indirect associations, mediation models were specified with parental mediation strategies as predictors, students’ overall digital device use score as the outcome, and parent–child interaction quality as the statistical mediator. Gender, grade level, place of residence, only-child status, primary caregiver, and household structure were entered as covariates.

Indirect effects were evaluated through nonparametric bootstrapping with 5,000 resamples, and statistical significance was inferred when the 95% bootstrap confidence interval did not include zero. In addition, structural equation modeling (SEM) via path analysis was used to examine the same set of associations within an integrated model. Model adequacy was judged using CFI, TLI, RMSEA, and SRMR. All tests were two-tailed with alpha set at 0.05. Given the cross-sectional design, the SEM results are interpreted as pattern estimates of association rather than evidence of temporal or causal ordering.

## 4 Results

To systematically address the research questions, the Results section is organized according to a sequential logic of digital device use profiles, differences in parental mediation, and potential pathway testing. Specifically, Sections 4.1–4.2 address Research Question 1, Sections 4.3–4.4 address Research Question 2, and Sections 4.5–4.6 further examine the mediating potential pathway proposed in Research Question 3.

### 4.1 Descriptive analysis of digital device use and parent–child conflict among primary and secondary school students

This section provides a comprehensive descriptive analysis of students’ digital device use characteristics across multiple dimensions, including device types, use time, use content, use contexts, use habits, and parent–child conflict related to digital device use.

#### 4.1.1 Types of digital devices used and personal ownership

**Figure 1:**
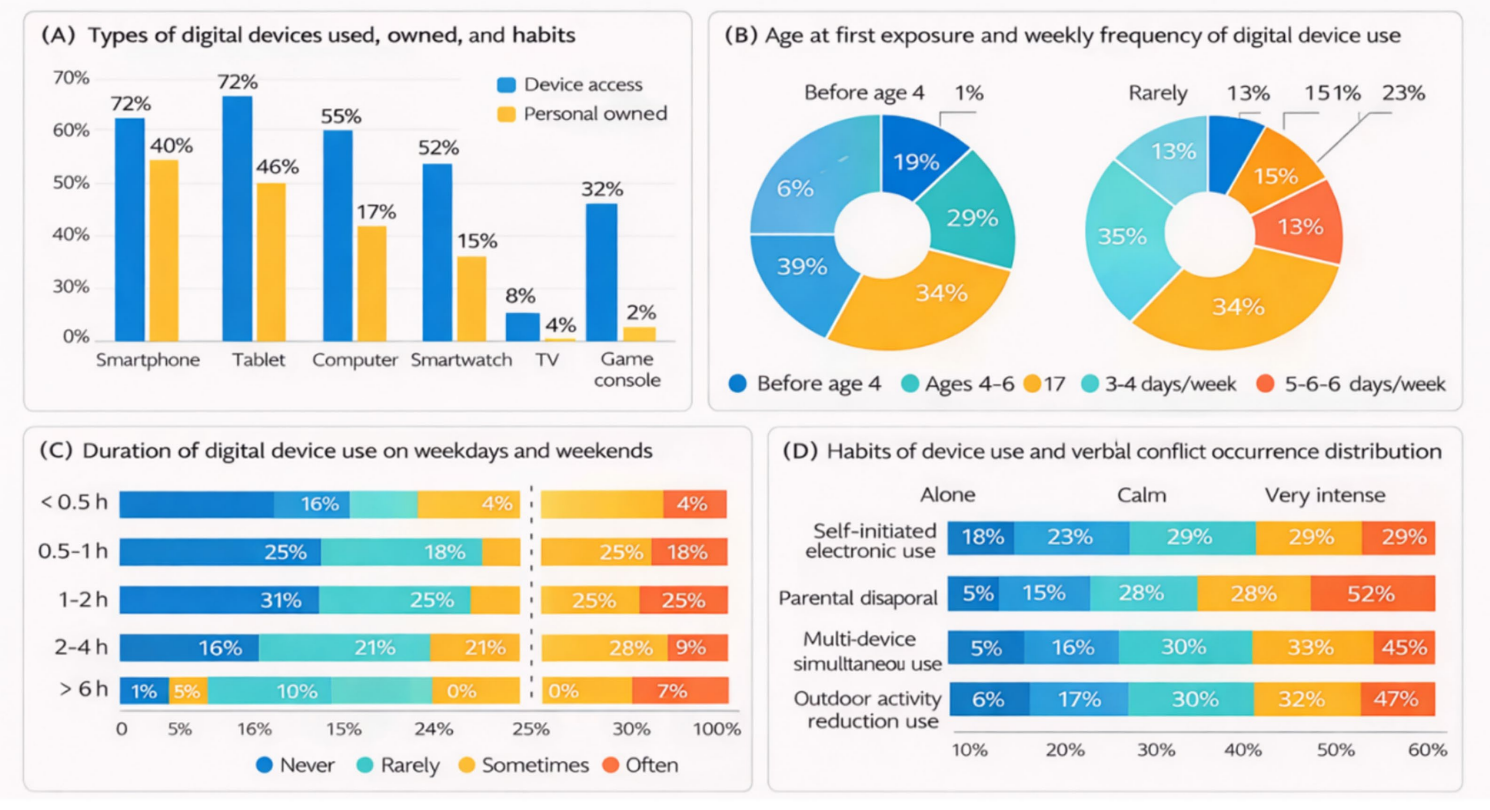
Digital device use profiles among primary and secondary school students. Percentages represent the proportion of students reporting each category. Panel **(A)** presents device access, personal ownership, and usage patterns. Panel **(B)** summarizes age of first exposure, usage habits, and family conflict frequency and intensity. Panel **(C)** compares duration of use between weekdays and weekends. Panel **(D)** illustrates self-reported habits of digital device use and verbal conflict occurrence distribution.

As shown in Figure 1A, the types of digital devices used by primary and secondary school students exhibited substantial diversity. Smartphones and tablet computers were the most commonly used devices, each reported by more than half of the sample. These were followed by television sets, smartwatches, and computers, whereas game consoles were used by a relatively small proportion of students. Overall, students’ digital device use was predominantly characterized by portable, multifunctional mobile devices, reflecting a clear preference for mobile digital media.

With regard to personal ownership, a considerable proportion of students reported owning at least one personal digital device, and some students owned multiple devices. Personally owned devices were primarily those with communication or learning functions, suggesting a high level of accessibility and privatization of digital devices in students’ daily lives.

#### 4.1.2 Patterns of digital device use time

In terms of the age at first exposure to digital devices (Figure 1B), students most commonly reported initial use during the preschool period or early primary school years, with relatively higher proportions observed in the 4–6 and 6–9 age ranges. This pattern indicates an increasingly early onset of digital device use among students.

Regarding frequency of use, most students reported using digital devices either “occasionally” or “1–2 days per week.” However, a non-negligible proportion reported using digital devices three or more days per week, highlighting variability in use frequency within the student population.

Patterns of use duration differed between weekdays and weekends (or holidays), as illustrated in Figure 1C. On weekdays, students’ total daily use time tended to cluster at shorter durations, whereas on weekends or holidays, overall use time was longer, with a higher proportion of students reporting extended use. In general, digital device use was more frequent and sustained on non-school days.

#### 4.1.3 Content of digital device use

As shown in Figures 1A,D, students’ digital device use content was mainly oriented toward learning, information acquisition, and entertainment. For learning- and information-related activities, a relatively high proportion of students reported engaging in such use “sometimes” or “often,” suggesting that digital devices have become an important resource for accessing information and learning materials.

In contrast, entertainment- and social-related uses exhibited pronounced individual differences. While some students reported limited or no engagement in online social interaction, others used digital media to varying degrees for communication or entertainment, reflecting selective and individualized patterns of content use.

#### 4.1.4 Contexts and habits of digital device use

As illustrated in Figure 1D, independent use of digital devices was common among students. Although simultaneous use of multiple devices or multitasking across different functions was observed, such behaviors were not predominant. In addition, most students did not report a substantial reduction in outdoor activities or peer interactions due to digital device use; nevertheless, a subset of students appeared to be affected to some extent, warranting further attention.

#### 4.1.5 Parent–child conflict related to digital device use

With respect to parent–child conflict associated with digital device use (Figure 1D), nearly half of the students reported no conflict with their parents during the reference period. Among those who did report conflict, occurrences were mainly occasional or low in frequency. Overall, parent–child conflict related to digital device use was generally of low to moderate intensity, although a small proportion of students reported experiencing relatively intense verbal conflicts, suggesting that digital device use may be associated with reduced interaction quality in some families.

Taken together, digital device use among primary and secondary school students was characterized by high prevalence, early initiation, and diverse use contexts. Substantial individual differences were observed in use patterns and related family interactions, providing an empirical basis for subsequent analyses of parental mediation strategies and their underlying potential pathway.

### 4.2 Differences in digital device use across sociodemographic variables

To examine whether students’ digital device use profiles differed across sociodemographic characteristics, group comparisons were conducted based on gender, grade level, place of residence, only-child status, primary caregiver, and household structure. Differences were examined across five dimensions: device type, use time, use content, use context, and use habits. The results of these comparisons are presented in Table 1.

**Table 1:** Differences in digital device use among primary and secondary school students across sociodemographic groups (M ± SD).

Grouping variable	Category (*N*)	Media type	Use time	Use content	Use context	Use habit	Statistic
Gender	Boys (619)	0.93 ± 0.21	2.59 ± 0.65	2.40 ± 0.65	2.57 ± 0.76	2.17 ± 0.85	*t* = −0.77–1.47
	Girls (616)	0.94 ± 0.18	2.57 ± 0.65	2.36 ± 0.65	2.54 ± 0.73	2.10 ± 0.80	ns
Grade	Grade 4 (218)	2.32 ± 0.78	0.93 ± 0.18	2.62 ± 0.64	2.36 ± 0.58	2.62 ± 0.72	*F* = 16.35**
	Grade 5 (232)	2.41 ± 0.83	0.90 ± 0.24	2.70 ± 0.66	2.56 ± 0.68	2.72 ± 0.75	
	Grade 6 (329)	1.95 ± 0.73	0.94 ± 0.17	2.47 ± 0.60	2.28 ± 0.60	2.47 ± 0.67	
	Grade 7 (275)	2.00 ± 0.81	0.93 ± 0.20	2.60 ± 0.64	2.33 ± 0.67	2.47 ± 0.78	
	Grade 8 (164)	2.05 ± 0.91	0.95 ± 0.18	2.54 ± 0.71	2.45 ± 0.66	2.54 ± 0.80	
Residence	Rural (297)	0.94 ± 0.18	2.64 ± 0.67	2.31 ± 0.61	2.56 ± 0.73	2.24 ± 0.85	*t* = −2.33*
	Urban (938)	0.93 ± 0.20	2.56 ± 0.64	2.41 ± 0.66	2.55 ± 0.76	2.10 ± 0.82	
Only-child status	Only child (529)	0.94 ± 0.19	2.56 ± 0.64	2.37 ± 0.66	2.55 ± 0.76	2.10 ± 0.81	ns
	Non-only child (706)	0.92 ± 0.20	2.60 ± 0.65	2.39 ± 0.64	2.56 ± 0.74	2.16 ± 0.84	
Primary caregiver	Parents (1018)	0.93 ± 0.20	2.56 ± 0.64	2.37 ± 0.64	2.54 ± 0.74	2.13 ± 0.82	*t* = −3.07*
	Grandparents (217)	0.95 ± 0.18	2.70 ± 0.68	2.44 ± 0.67	2.61 ± 0.79	2.18 ± 0.88	
Household type	Nuclear family (702)	0.93 ± 0.20	2.57 ± 0.64	2.39 ± 0.65	2.54 ± 0.71	2.14 ± 0.81	*F* = 0.37–2.89
	Extended family (373)	0.94 ± 0.19	2.56 ± 0.61	2.36 ± 0.64	2.55 ± 0.78	2.15 ± 0.84	ns
	Other (160)	0.93 ± 0.20	2.70 ± 0.74	2.40 ± 0.67	2.62 ± 0.84	2.11 ± 0.89	ns

Values are presented as mean ± standard deviation (M ± SD). Independent-samples *t*-tests were used for two-group comparisons, and one-way analyses of variance (ANOVA) were conducted for comparisons involving more than two groups. Media type, use time, use content, use context, and use habit represent composite scores of corresponding digital device use dimensions. *p* < 0.05; *p* < 0.01; ns = not significant.

#### 4.2.1 Gender differences

As shown in Table 1, independent-samples t tests indicated that there were no statistically significant differences between boys and girls across the five dimensions of digital device use—device type, use time, use content, use context, and use habits (all *p* > 0.05). These results suggest that primary and secondary school students of different genders exhibited largely comparable digital device use profiles.

#### 4.2.2 Grade-level differences

Significant differences in digital device use were observed across grade levels on several dimensions (see Table 1). One-way analyses of variance revealed significant main effects of grade on device type (*F* = 16.35, *p* < 0.01), use content (*F* = 4.71, *p* < 0.01), use context (*F* = 7.36, *p* < 0.01), and use habits (*F* = 5.19, *p* < 0.01). In contrast, differences in use time across grade levels did not reach statistical significance (*p* > 0.05).

Descriptive statistics further indicated variability in patterns of device access, content engagement, and use contexts across grades, suggesting that grade level was closely associated with specific characteristics of digital device use.

#### 4.2.3 Differences by place of residence

With respect to place of residence, significant differences were observed between rural and urban students on selected dimensions of digital device use (see Table 1). *t* tests showed that rural and urban students differed significantly in use content (*t* = −2.33, *p* < 0.05) and use habits (*t* = 2.43, *p* < 0.05). No statistically significant differences were found for device type, use time, or use context (all *p* > 0.05).

#### 4.2.4 Only-child status

As presented in Table 1, independent-samples *t* tests indicated that students who were only children and those with siblings did not differ significantly across the five dimensions of digital device use—device type, use time, use content, use context, and use habits (all *p* > 0.05). These findings suggest that only-child status was not associated with meaningful differences in students’ digital device use profiles.

#### 4.2.5 Differences by primary caregiver

Differences associated with primary caregiver status were examined by comparing students primarily cared for by their parents with those cared for by grandparents. As shown in Table 1, a statistically significant difference was found for use time (*t* = −3.07, *p* < 0.05), with students under grandparental care reporting higher levels of digital device use time than those primarily cared for by parents. No significant differences were observed for device type, use content, use context, or use habits (all *p* > 0.05).

#### 4.2.6 Differences by household structure

One-way analyses of variance were conducted to examine differences across household structure (nuclear families, extended families, and other family types). The results indicated that household structure was not significantly associated with differences in device type, use time, use content, use context, or use habits (all *p* > 0.05; see Table 1).

### 4.3 Parental mediation in students’ digital device use

#### 4.3.1 Descriptive statistics of parental mediation strategies

Parental mediation strategies were examined across five dimensions—co-use, active mediation, restriction, monitoring, and technical restriction—among students who reported using digital devices. Descriptive statistics summarizing the distribution of parental mediation strategies are presented in Table 2.

**Table 2:** Descriptive statistics of parental mediation strategies in students’ digital device use.

Dimension	M	SD
Co-use	2.13	0.66
Active mediation	2.31	0.71
Restriction	3.16	0.65
Monitoring	1.72	0.82
Technical restriction	1.82	0.83

As shown in Table 2, among the five parental mediation strategies, restriction exhibited the highest mean score (M = 3.16, SD = 0.65), followed by active mediation (M = 2.31, SD = 0.71) and co-use (M = 2.13, SD = 0.66). In contrast, monitoring (M = 1.72, SD = 0.82) and technical restriction (M = 1.82, SD = 0.83) showed relatively lower mean levels. Overall, parental mediation in the context of students’ digital device use was characterized primarily by behavioral restriction, whereas direct monitoring and technology-based approaches were less frequently reported.

#### 4.3.2 Co-use strategies

**Figure 2:**
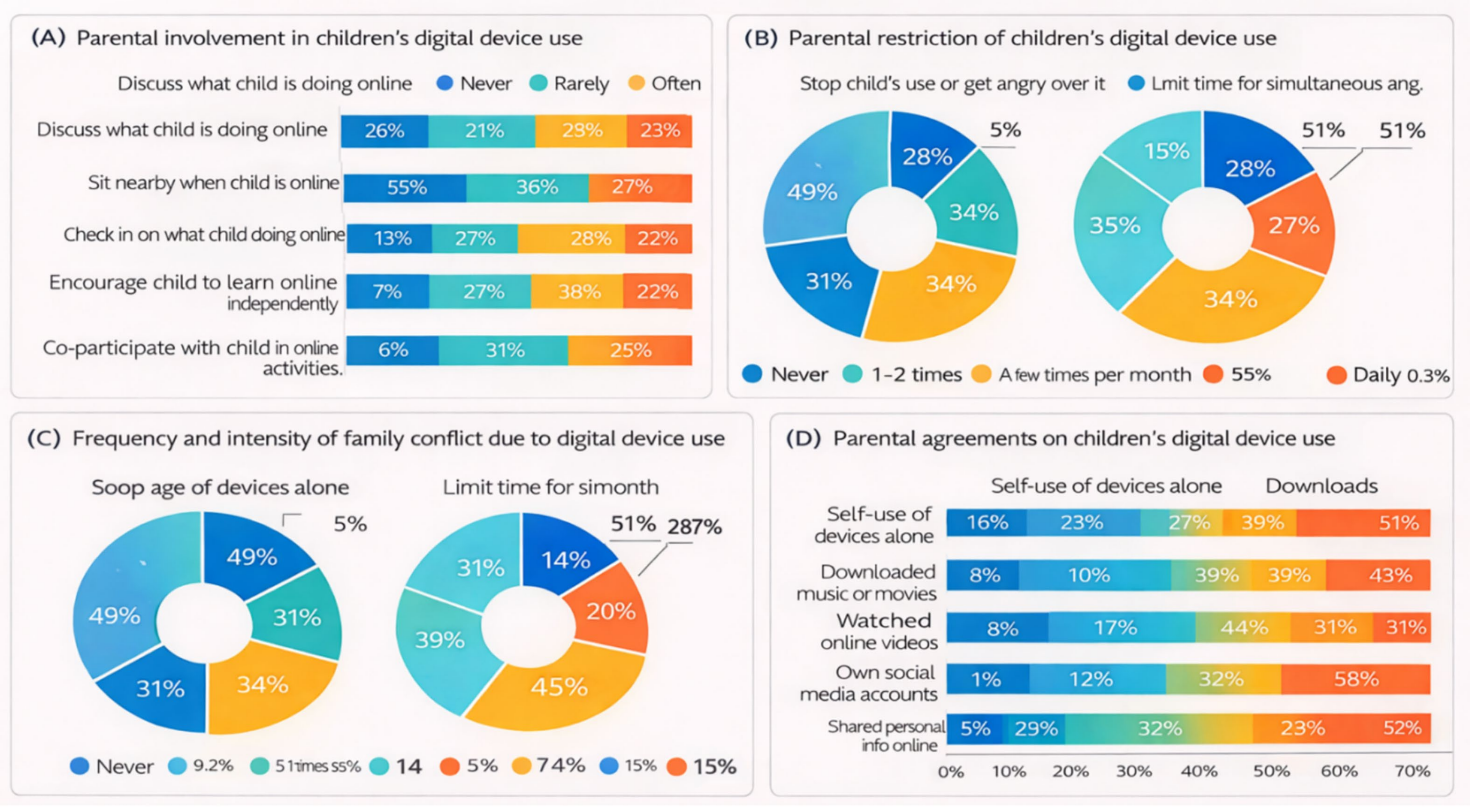
Digital device use and family context among primary and secondary school students. Percentages represent the proportion of students reporting each category. Panel **(A)** presents parental involvement in children’s digital device use, Panel **(B)** shows parental restriction of children’s digital device use, Panel **(C)** depicts the frequency and intensity of family conflict due to digital device use, and Panel **(D)** displays parental agreements on children’s digital device use.

Co-use strategies primarily involve interactive parental engagement during children’s digital device use, such as discussing online content with children or remaining physically nearby while devices are being used. As illustrated in Figure 2A, approximately 49.64% of parents reported “sometimes” discussing online content with their children, whereas about 22.91% reported “never” engaging in such discussions. With regard to staying nearby while children use digital devices, a relatively higher proportion of parents reported “never,” indicating variability in the level of parental presence during device use.

#### 4.3.3 Active mediation strategies

Active mediation strategies include providing assistance when children encounter online difficulties, explaining the quality and credibility of online information, and offering guidance related to online safety. As shown in Figure 2A, many parents reported a moderate level of involvement in explaining online safety and information quality. However, the proportion of parents who reported “often” or “always” providing help when children encountered online problems was relatively low, suggesting variability in the consistency of active parental support.

#### 4.3.4 Restrictive mediation strategies

Restrictive mediation strategies refer to parental limitations placed on children’s digital device use behaviors and content, including instant messaging, downloading media, watching online videos, social media use, and the sharing of personal information (Figure 2B). The frequency and intensity of family conflict related to digital device use are shown in Figure 2C. Parental agreements on children’s digital device use, particularly those related to personal privacy protection, are presented in Figure 2D and were especially prominent. A relatively high proportion of parents reported limiting behaviors such as uploading personal information or sharing personal details with others. In contrast, restrictions on routine entertainment or communication activities were more unevenly distributed.

#### 4.3.5 Monitoring strategies

Monitoring strategies involve parents checking children’s browsing histories, instant messaging records, or social media accounts. As shown in Figure 2D, a substantial proportion of parents reported “never” engaging in such monitoring behaviors. For example, 47.77% of parents indicated that they never checked their children’s browsing records, suggesting generally low levels of direct parental monitoring of digital activities.

#### 4.3.6 Technical restriction strategies

Technical restriction strategies primarily involve the use of software or technical tools to filter online content, monitor browsing activities, or limit time spent online. As illustrated in Figure 2D, more than half of parents reported not using technical tools to filter inappropriate websites or check browsing records. Nevertheless, a proportion of parents reported using technical measures to limit online time or to protect against spam and viruses, indicating differential application of technical restrictions depending on specific functional goals.

### 4.4 Differences in parental mediation strategies across sociodemographic groups

To examine differences in parental mediation strategies across sociodemographic characteristics, group comparisons were conducted based on gender, grade level, place of residence, only-child status, primary caregiver, and household structure. Differences were analyzed across five dimensions of parental mediation: co-use, active mediation, restriction, monitoring, and technical restriction. The results of these comparisons are presented in Table 3.

**Table 3:** Parental mediation of children’s digital device use across sociodemographic groups (M ± SD).

Grouping Variable	Category (*N*)	Co-use	Active mediation	Restriction	Monitoring	Technical restriction	Statistic
Overall	Total sample	2.13 ± 0.66	2.31 ± 0.71	3.16 ± 0.65	1.72 ± 0.82	1.82 ± 0.83	—
Gender	Boys (619)	2.13 ± 0.65	2.26 ± 0.73	3.19 ± 0.65	1.72 ± 0.82	1.83 ± 0.86	*t* = −2.18*
	Girls (616)	2.13 ± 0.66	2.35 ± 0.69	3.13 ± 0.65	1.72 ± 0.80	1.80 ± 0.81	
Grade	Grade 4 (218)	2.31 ± 0.59	2.40 ± 0.64	3.13 ± 0.62	1.87 ± 0.76	1.92 ± 0.85	*F* = 3.27*–13.40**
	Grade 5 (232)	2.12 ± 0.63	2.30 ± 0.65	2.97 ± 0.67	1.77 ± 0.81	1.77 ± 0.79	
	Grade 6 (329)	2.10 ± 0.66	2.23 ± 0.74	3.32 ± 0.56	1.58 ± 0.80	1.80 ± 0.85	
	Grade 7 (275)	2.08 ± 0.71	2.29 ± 0.77	3.25 ± 0.62	1.69 ± 0.82	1.79 ± 0.83	
	Grade 8 (164)	2.09 ± 0.64	2.43 ± 0.69	3.04 ± 0.75	1.77 ± 0.86	1.85 ± 0.85	
Residence	Rural (297)	2.19 ± 0.65	2.30 ± 0.64	3.22 ± 0.58	1.80 ± 0.76	1.81 ± 0.78	*t* = 2.02*
	Urban (938)	2.11 ± 0.66	2.31 ± 0.73	3.15 ± 0.67	1.84 ± 0.83	1.81 ± 0.85	
Only-child status	Only child (529)	2.18 ± 0.68	2.35 ± 0.72	3.16 ± 0.65	1.76 ± 0.84	1.82 ± 0.85	*t* = 2.17*
	Non-only child (706)	2.10 ± 0.64	2.28 ± 0.70	3.16 ± 0.65	1.69 ± 0.79	1.82 ± 0.82	
Primary caregiver	Parents (1018)	2.15 ± 0.65	2.34 ± 0.71	3.18 ± 0.64	1.74 ± 0.82	1.83 ± 0.85	*t* = 2.47*–3.02**
	Grandparents (217)	2.03 ± 0.65	2.18 ± 0.71	3.09 ± 0.69	1.63 ± 0.77	1.75 ± 0.77	
Household type	Nuclear (702)	2.15 ± 0.65	2.32 ± 0.71	3.19 ± 0.64	1.73 ± 0.81	1.82 ± 0.82	*F* = 0.27–2.12
	Extended (373)	2.12 ± 0.65	2.31 ± 0.69	3.14 ± 0.63	1.73 ± 0.84	1.83 ± 0.87	ns
	Other (160)	2.07 ± 0.67	2.27 ± 0.76	3.08 ± 0.74	1.64 ± 0.78	1.77 ± 0.79	ns

Values are presented as mean ± standard deviation (M ± SD). Independent-samples *t*-tests were conducted for two-group comparisons, and one-way analyses of variance (ANOVA) were used for comparisons involving more than two groups. Co-use, active mediation, restriction, monitoring, and technical restriction represent dimensions of parental mediation of children’s digital device use. *p* < 0.05; *p* < 0.01; ns = not significant.

#### 4.4.1 Gender differences

As presented in Table 3, independent-samples t tests indicated that no statistically significant gender differences were observed across four dimensions of parental mediation—co-use, restriction, monitoring, and technical restriction (all *p* > 0.05). However, a significant gender difference emerged for active mediation (*t* = −2.18, *p* < 0.05), with girls reporting higher levels of active mediation than boys.

#### 4.4.2 Grade-level differences

Differences in parental mediation strategies across grade levels are summarized in Table 3. One-way analyses of variance revealed significant main effects of grade on active mediation (*F* = 3.27, *p* < 0.05) and restriction (*F* = 13.40, *p* < 0.01). No significant grade-level differences were found for co-use, monitoring, or technical restriction (all *p* > 0.05). Descriptive statistics suggested variability across grades in the extent to which parents adopted active mediation and restrictive strategies.

#### 4.4.3 Differences by place of residence

As shown in Table 3, a significant difference between rural and urban students was observed for the restriction dimension (*t* = 2.02, *p* < 0.05), with higher restriction scores reported among rural students. No statistically significant differences were found for co-use, active mediation, monitoring, or technical restriction (all *p* > 0.05).

#### 4.4.4 Differences by only-child status

With respect to only-child status, independent-samples t tests indicated a significant difference in co-use (*t* = 2.17, *p* < 0.05), with higher co-use scores reported for only children compared with students with siblings. No significant differences were found for active mediation, restriction, monitoring, or technical restriction (all *p* > 0.05), as shown in Table 3.

#### 4.4.5 Differences by primary caregiver

As presented in Table 3, significant differences were observed between students primarily cared for by parents and those under grandparental care for both co-use (*t* = 2.47, *p* < 0.05) and active mediation (*t* = 3.02, *p* < 0.01). Students cared for by parents reported higher levels of co-use and active mediation. No significant differences were found for restriction, monitoring, or technical restriction (all *p* > 0.05).

#### 4.4.6 Differences by household structure

One-way analyses of variance examining household structure (nuclear families, extended families, and other family types) indicated no statistically significant differences across any of the five parental mediation dimensions—co-use, active mediation, restriction, monitoring, and technical restriction (all *p* > 0.05), as shown in Table 3.

As a preliminary diagnostic, the common-method assessment did not indicate that a single general factor dominated item covariance, which provides some support for the interpretability of the subsequent association models. This should nevertheless be viewed as a limited diagnostic rather than definitive evidence that method effects were absent.

### 4.5 Associations between digital device use and parental mediation strategies

Pearson correlation analyses were conducted to examine the associations between students’ digital device use and parental mediation strategies. The results are presented in Table 4.

**Table 4:** Correlations between digital device use and parental mediation strategies.

Variable	Device type	Use time	Use content	Use context	Use habits
Co-use	0.02	0.03	0.15**	0.19**	0.14**
Active mediation	0.02	−0.01	0.14**	0.10**	−0.04
Restriction	−0.14**	−0.32**	−0.57**	−0.50**	−0.40**
Monitoring	−0.03	−0.03	0.03	0.09**	0.09**
Technical restriction	−0.02	−0.04	0.07*	0.07**	0.08**

*p < 0.05; **p < 0.01.

Overall, restriction was negatively associated with device type, use time, use content, use context, and use habits. Use content showed positive associations with co-use, active mediation, and technical restriction, and a negative association with restriction. Use context was positively associated with co-use, active mediation, monitoring, and technical restriction, and negatively associated with restriction. Use habits were positively associated with co-use, monitoring, and technical restriction, and negatively associated with restriction.

### 4.6 Testing the statistical mediation of parent–child interaction quality

To evaluate the proposed relational pathway, mediation models were estimated with parent–child interaction quality positioned as the statistical mediator between parental mediation strategies and students’ overall digital device use. Because the data were cross-sectional, the analysis was intended to test indirect association patterns rather than causal mechanisms.

In these models, parental mediation strategies were entered as independent variables, students’ overall digital device use scores were treated as the dependent variable, and parent–child interaction quality served as the mediator. Gender, grade level, place of residence, only-child status, primary caregiver, and household structure were included as control variables. Bootstrapping procedures with 5,000 resamples were applied, and indirect association were considered statistically significant when the 95% confidence intervals did not include zero.

As shown in Table 5, parent–child interaction quality was a significant statistical mediator in the models for co-use, active mediation, and restrictive mediation.

**Table 5:** Indirect associations via parent–child interaction quality in the relationship between parental mediation strategies and students’ digital device use (bootstrap, *N* = 1,235).

Parental mediation strategy	Direct effect (c′)	Indirect effect (a × b)	95% CI (bootstrap)	Mediation type
Co-use	0.08*	0.05*	[0.02, 0.09]	Partial mediation
Active mediation	0.10*	0.06*	[0.03, 0.10]	Partial mediation
Restriction	−0.31**	−0.18**	[−0.24, −0.12]	Partial mediation
Monitoring	0.04	0.02	[−0.01, 0.05]	Not significant
Technical restriction	0.05	0.03	[−0.01, 0.06]	Not significant

Values represent standardized regression coefficients. Bootstrap resampling was conducted with 5,000 iterations. All models controlled for gender, grade, residence, only-child status, primary caregiver, and household type. * *p* < 0.05, ** *p* < 0.01.

For co-use, the indirect association through better parent–child interaction quality was statistically significant (a × b = 0.05, 95% CI [0.02, 0.09]). The direct path remained significant after the mediator was included, indicating partial statistical mediation.

Active mediation showed a similar pattern. Its indirect association via parent–child interaction quality was statistically significant (a × b = 0.06, 95% CI [0.03, 0.10]), again consistent with partial statistical mediation rather than a fully mediated pathway.

Restrictive mediation displayed both a significant direct negative association with students’ digital device use (c’ = −0.31, *p* < 0.01) and a significant indirect association through parent–child interaction quality (a × b = −0.18, 95% CI [−0.24, −0.12]). This suggests that rule-based mediation was related to device use both directly and through the relational atmosphere in which rules were implemented.

By contrast, the indirect paths for monitoring and technical restriction were not statistically significant because their bootstrap confidence intervals included zero. In other words, surveillance-oriented and technical strategies were not reliably linked with digital device use through parent–child interaction quality in the present sample.

Overall, these results support the view that parent–child interaction quality may serve as an important relational pathway for some, but not all, parental mediation strategies. Given the cross-sectional nature of the data, these indirect paths should be interpreted as statistical associations rather than causal mechanisms.

## 5 Discussion

### 5.1 Key findings and interpretation

This study provides cross-sectional evidence that parental mediation strategies are differentially associated with students’ digital device use and with the quality of parent–child interaction. Restrictive mediation was linked with lower use time, less entertainment-oriented use, and less intensive device-use habits, whereas co-use and active mediation were more closely associated with better parent–child interaction quality. These findings suggest that family media management contains at least two distinguishable elements: behavioral regulation and relational engagement.

From a family-systems perspective, this pattern is understandable. Rules about device use are implemented inside an ongoing interaction system, not in a social vacuum. Restrictive mediation may be associated with lower use simply because it places clearer behavioral boundaries on children’s access. By contrast, co-use and active mediation appear to operate more through conversation, explanation, and shared attention, which may strengthen openness and reduce friction in everyday parent–child exchanges. The findings therefore add nuance to the parental mediation literature by showing that different strategies are linked to different facets of children’s digital-device experience.

### 5.2 The mediating role of parent–child interaction quality

A key contribution of the study is the finding that parent–child interaction quality functioned as a statistically significant relational link in several of the tested models. This result is theoretically meaningful because it connects family systems theory with parent–child communication theory: parental mediation is not merely a set of isolated control techniques, but part of a broader interaction pattern through which children interpret rules, support, and guidance.

More specifically, the results suggest that when parental mediation is embedded in warmer, clearer, and more communicative day-to-day interactions, its association with children’s digital device use may look different than when mediation is carried out in a distant or conflict-laden relational climate. In this sense, parent–child interaction quality may help explain why strategies that appear similar on the surface do not always show identical associations in empirical models. At the same time, because the data are cross-sectional, the current study cannot determine whether better interaction quality precedes more effective mediation, whether the reverse is true, or whether both are shaped by third variables such as child temperament or parenting stress.

### 5.3 Implications for family education practice

The findings have several implications for family education practice. First, families may benefit from viewing digital-device guidance as both a rule-setting task and a relationship task. Clear limits remain relevant, particularly for time use and entertainment-oriented content, but rules may be more sustainable when they are accompanied by explanation, shared discussion, and consistent communication.

For schools and practitioners, the results point to the value of parent guidance programs that go beyond technical restriction alone. Workshops or family education initiatives can help parents combine reasonable boundaries with discussion-based mediation, shared use, and conflict management. Such programs may be especially useful in families where grandparents are primary caregivers or where routine supervision is more difficult, because those contexts may require more deliberate support for communication and coordinated family media rules.

### 5.4 Limitations and directions for future research

Several limitations should be acknowledged. First, the study used a cross-sectional design, so temporal ordering and causal inference cannot be established. Second, although students and parents reported different parts of the questionnaire, the study still relied on self-report data, which may be affected by recall bias and social desirability. Third, the sample came from one district in Sichuan Province, which limits broader generalizability. In addition, some potentially important background indicators - including exact age, household socioeconomic status, and caregivers’ marital status - were not available for the present analyses. Fourth, the current models focused on one relational pathway, namely parent–child interaction quality, and did not test other plausible explanatory variables such as parenting stress, child self-control, or school-level digital norms. Future studies should use longitudinal or multi-wave designs, draw on more diverse regional samples, include richer family background variables, and compare alternative mediation or moderation models.

## 6 Conclusion

This study examined how parental mediation strategies were associated with digital device use among primary and secondary school students and whether parent–child interaction quality statistically linked those associations. Using paired reports from students and parents, the study found that restrictive mediation was associated with lower levels of device use, whereas co-use and active mediation were more strongly associated with better parent–child interaction quality.

Taken together, the findings suggest that children’s digital-device practices should be understood within a family relational context rather than solely as an individual behavior-management problem. Parent–child interaction quality appears to be an important correlational pathway in the association between parental mediation and students’ digital device use, although the direction of that pathway requires further longitudinal verification.

## Data Availability

The raw data supporting the conclusions of this article will be made available by the authors, without undue reservation.
